# pH-Dependent Foam Formation Using Amphoteric Colloidal Polymer Particles

**DOI:** 10.3390/polym12030511

**Published:** 2020-02-27

**Authors:** Sayaka Fukui, Tomoyasu Hirai, Yoshinobu Nakamura, Syuji Fujii

**Affiliations:** 1 Department of Applied Chemistry, Faculty of Engineering, Osaka Institute of Technology, 5-16-1 Omiya, Asahi-ku, Osaka 535-8585, Japan; e1516098@st.oit.ac.jp (S.F.); tomoyasu.hirai@oit.ac.jp (T.H.); yoshinobu.nakamura@oit.ac.jp (Y.N.); 2 Nanomaterials Microdevices Research Center, Osaka Institute of Technology, 5-16-1 Omiya, Asahi-ku, Osaka 535-8585, Japan

**Keywords:** pH-sensitive, polymer particle, foam, bubble, adsorption, interface, moiré pattern

## Abstract

Near-monodispersed micrometer-sized polystyrene (PS) particles carrying amidino and carboxyl groups on their surfaces were synthesized by soap-free emulsion polymerization using an amphoteric free radical initiator. The resulting amphoteric PS particles were characterized in terms of diameter, morphology, disperibility in aqueous media and surface charge using scanning electron microscopy (SEM), optical microscopy (OM), sedimentation rate and electrophoretic measurements. At pH 2.0, where the amidino groups are protonated (positively charged), and at pH 11.0, where the carboxyl groups are deprotonated (negatively charged), the PS particles were well dispersed in aqueous media via electrostatic repulsion. At pH 4.8, where the surface charges are neutral, the PS particles were weakly aggregated. Furthermore, it was confirmed that the PS particles can function as a pH-sensitive foam stabilizer: foamability and foam stability were higher at pH 2.0 and 4.8, where the PS particles can be adsorbed to the air–water interface, and lower at pH 11.0, where the PS particles tend to disperse in bulk aqueous medium. SEM and OM studies indicated that hexagonally close-packed arrays of PS particles were formed on the bubble surfaces and moiré patterns were observed on the dried foams. Moreover, the fragments of dried foams showed iridescent character under white light.

## 1. Introduction

Solid particles are known to be adsorbed to the air–water interface to stabilize aqueous foams [1,2,3,4,5]. Foams stabilized with inorganic particles (e.g., silica [6,7], alumina [8] and graphene [9]) have been studied for a long time, and these days those stabilized with organic particles, including synthetic polymer particles [10,11,12,13,14] and natural particles (e.g., aquatic hyphomycete spores [15] and bacterial cells [16]) have started to gain interest. The synthetic polymer particles with specific surface chemistries can be designed using various functional monomers, initiators and colloidal stabilizers and by post surface modifications, which makes them attractive foam stabilizers. Thanks to this advantage, recently, there has been increasing interest in polymer particle-stabilized foams whose foamability, foam stability and microstructures can be controlled by external stimulus [17,18,19]. Understanding the foamation and defoamation phenomena could greatly contribute to develop food and ore floatation science and industry, where the particle-stabilized foams play crucial roles. By now, particles carrying pH-responsive acidic or basic functional groups have been utilized to prepare pH-sensitive aqueous foams. It has been demonstrated that polystyrene (PS) particles carrying poly[2 -(dimethylamino)ethyl methacrylate] [20], poly[2-(diethylamino)ethyl methacrylate] (PDEA) [21,22,23], poly(4-vinylpyrridine) [24] or poly(acrylic acid) [25] colloidal stabilizer on their surfaces can work as the pH-responsive particulate bubble stabilizer: the foams with high foamability and stability are prepared at pHs where the colloidal stabilizers are hydrophobic, and unstable/no foams are obtained at pHs where the colloidal stabilizers are highly hydrophilic. The polymer particles studied as a pH-sensitive foam stabilizer until now carry a single kind of pH-responsive functional groups with single p*K*_a_ values. Considering that the particle-stabilized foams are used at various pHs and in order to widen the application range of the foams, it is important to study on foams stabilized with polymer particles carrying functional groups with multiple p*K*_a_ values.

In the present study, first, we synthesized near-monodispersed micrometer-sized PS particles carrying amphoteric groups on their surfaces by soap-free emulsion polymerization using 2,2′-azobis[*N*-(2-carboxyethyl)-2-methylpropionamidine] tetrahydrate (ACMPA) as a free-radical initiator. Then, the effects of pH of aqueous media, where the particles were dispersed, on foamability and foam stability were investigated. The naked eye, and optical microscopy (OM) and scanning electron microscopy (SEM) were utilized to characterize the foamability, foam stability and their microstructures.

## 2. Materials and Methods

Free radical soap-free emulsion polymerization of styrene using ACMPA was conducted to synthesize PS particles carrying amidino and carboxyl groups (ACMPA-PS particles), referring to the method previously reported [26,27,28,29]. Briefly, styrene (10 g, Sigma-Aldrich, St Louis, MO, USA) and ACMPA (0.5 g, Wako Pure Chemical Industries, Ltd., Osaka, Japan) were mixed with aqueous medium (100 g, pH 10 adjusted using NaOH aqueous solution) for 10 min, and then the mixture was heated to 60 °C with magnetic stirring. The polymerization was conducted for 24 h under a nitrogen atmosphere. Successive centrifugation-redispersion cycles were used to purify the dispersion, with each supernatant being decanted and replaced with deionized water using a centrifuge (Hitachi CF16RX II type centrifuge with a Hitachi T15A 36 rotor, Tokyo, Japan). Particle size distribution was obtained by a Malvern Mastersizer 2000 instrument equipped with a small volume Hydro 2000SM sample dispersion unit. Zeta potentials were estimated from the electrophoretic mobility, measured at various pHs using a Malvern Zetasizer Nano ZS with a MPT-2 Autotitrator. Aqueous dispersions of the PS particles with pH of 2.0, 4.8 and 11.1 were shaken 100 times for 30 s with an amplitude of 30 cm by hand. Dispersibility of the PS particles and morphology and size of the bubbles were studied in a wet state by OM (Motic BA200, Shimadzu, Kyoto, Japan). Morphology and size of the PS particles and the bubble microstructures were examined in a dry state by SEM (VE-8800 instrument, Keyence, Osaka, Japan). The contact angle of the PS particles at the air–water interface was evaluated by direct observation after superglue vapor treatment [30,31,32,33,34].

## 3. Results and Discussion

### 3.1. Synthesis and Characterization of Amphoteric Polystyrene (PS) Particles

Free radical soap-free emulsion polymerization of styrene using ACMPA successfully led to production of the PS particles carrying amidino and carboxyl groups [26,27,28,29]. The ACMPA initiator carries both amidino and carboxyl groups, whose p*K*_a_ values are 3.6 and 9.8, respectively, and these polar functional groups are expected to appear on the particle surfaces [29,35]. The free radical soap-free emulsion polymerization has a benefit to be able to synthesize near-monodispersed particles with clean surfaces without molecular-level small surfactant. Six sedimentation/redispersion cycles using deionized water (pH not controlled) were conducted to remove NaOH, the initiator and its by-products in continuous polymerization medium. The PS particles were characterized in detail regarding their size, zeta potential and dispersibility using OM, SEM, and laser diffraction particle size analysis and electrophoretic mobility measurement. OM studies of aqueous dispersions after the polymerization indicated production of colloidally stable PS particles with no flocs. Polar amidino and carboxyl groups arising from the ACMPA should confer colloidal stability. The volume-average diameter was determined to be 1.1 ± 0.2 μm by laser diffraction particle size analysis. SEM observation indicated the production of spherical PS particles with the number-average particle diameter (*D*_n_) of 1.0 ± 0.1 μm (*n* = 100) (Figure S1, Supplementary Materials).

**Figure 1:**
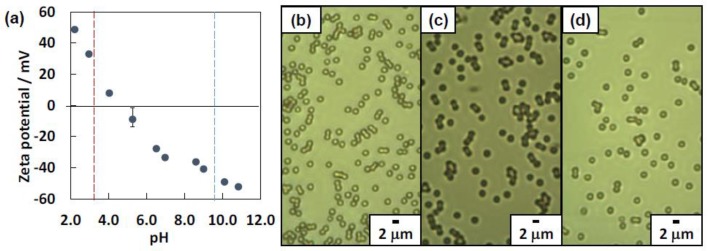
(**a**) Relationship between pH and zeta potential measured for the polystyrene particles carrying amidino and carboxyl groups (ACMPA-PS). (**b**–**d**) Optical micrographs of aqueous dispersions of ACMPA-PS particles observed at (**b**) pH 2.0, (**c**) pH 4.8 and (**d**) pH 11.1.

Dispersibility of the PS particles was studied at pH 2.0, 4.8 and 11.0 using OM (Figure 1b–d). At pH 2.0 and 11.0, colloidally stable particles were observed, whereas a few micrometer-sized flocs were observed in addition to colloidally stable particles at pH 4.8. Zeta potential measurements were conducted as a function of pH to verify the pH-sensitive surface charging nature of the PS particles (Figure 1a). The isoelectric point was determined to be 4.6, which was similar with the value reported by Nagao et al. [29]. The PS particle surface was positively charged due to protonation of the amidino groups on the particle surface, which were originated from the ACMPA initiator, below pH 4.6. The zeta potential was +48 ±1 mV at pH 2.0 and decreased with an increase of pH. Above pH 4.6, the zeta potentials were negative and showed a drop at ca. pH = 9, which is near the p*K*_a_ value of carboxyl groups of the ACMPA, due to deprotonation of the carboxyl groups on the particle surfaces. There is a possibility that the negative charges also originated from a hydroxide anion adsorbed on the particle surfaces [36,37]. The zeta potential results confirmed that surface charge density on the PS particles, in other words the surface hydrophilicity–hydrophobicity balance, can be tuned by pH.

Sediment height (*H*_s_) as a function of time was investigated using 5.0 wt % aqueous dispersions at pH 2.0, 4.8 and 11.1 (Figure S2, Supplementary Materials). The PS particles sediment a little bit faster at pH 4.8, where the particles were weakly flocculated, than those at pH 2.0 and 11.1, where the particles are in a colloidally stable state. Based on the sedimentation rates ($-\frac{dH_{s}}{dt}$), the particle sizes were calculated using the equations shown below [32,38].

(1) $$V_{0}\left(d\right)=-\frac{dH_{s}}{dt}\frac{1+K\phi /{\left(1-\phi \right)}^{3}}{1-\phi }$$

and

(2) $$V_{0}\left(d\right)=\frac{\Delta \rho gd^{2}}{18\eta }$$

where $V_{0}\left(d\right)$ is the rate of sedimentation at infinite dilution, K is 4.6, ϕ is the particle volume fraction in the dispersion (0.0476), Δρ is the density difference between aqueous phase and the PS particle (0.05 × 10^6^ g m^−3^), g is the gravitational acceleration (9.8 m s^−2^), d is the particle diameter, and η is the viscosity of aqueous phase (1.01 g m^−1^ s^−1^). The particle diameter was estimated to be 1.46 μm at pH 4.8, which was a little bit larger than those estimated at pH 2.0 (1.40 μm) and pH 11.1 (1.27 μm). From this result, the formation of PS particle flocs at pH 4.8 can be confirmed. It was surprising that the PS particles did not form large aggregates near the isoelectric point. The reason is unclear, but solvated PS hairy layers might be formed on the particle surface due to polar polymer end groups, which could work as a steric stabilizer [39]. The *H*_s_ values determined 1 month after start of sedimentation experiment were 2.5, 3.2 and 2.0 mm at pH 2.0, 4.8 and 11.1, respectively. At pH 2.0 and 11.1, the PS particles sediment in an individually dispersed state and form a closely-packed structure. On the other hand, the PS particles formed sediment containing water voids generated due to flocculated particles [32].

### 3.2. Foams Stabilized with Amphoteric PS Particles

**Figure 2:**
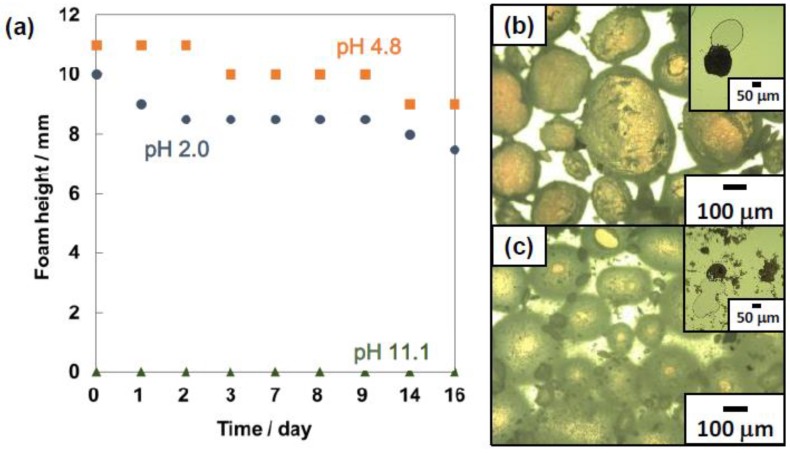
(**a**) Temporal change of foam height observed for the foams prepared at pH 2 (●), pH 4.8 (■), pH 11.1 (▲). (**b**,**c**) Optical micrographs of bubbles stabilized with ACMPA-PS particles prepared at (**b**) pH 2.0 and (**c**) 4.8. Insets of (**b**,**c**) show the bubbles between glass substrates after the application of pressure. An inset of (**b**,**c**) shows the bubbles after the application of pressure between glass substrates.

Foamability and foam stability are known to depend on surface hydrophilic-hydrophobic balance of the particles [2,3,4,5]. The heights of foam layers, which were formed on the upper part of aqueous dispersion of the particles due to creaming, were measured at 25 °C using a ruler after hand shaking the aqueous particle dispersions (5.0 wt %, 3.0 mL, pH 2.0, 4.8 and 11.1) and air in a 13.5 mL glass vial with a screw cap to evaluate foamability and foam stability (Figure 2a).

The pH of the dispersions were adjusted using aqueous solutions of either HCl or NaOH. When the planar aqueous dispersion–air surface appeared, the foam height was defined to be 0 mm. At pH 2.0 and 4.8, the foam layers were stable for over 16 days. At pH 4.8, the surface charge of the PS particles are neutral and the hydrophobic PS particles could be adsorbed to the air–water interface to stabilize the foams. The PS particles with positive surface charge at pH 2.0 could be electrostatically adsorbed to the anionic air–water interface [40] and worked as a foam stabilizer. The foamability at pH 2.0 was a little bit lower than that at pH 4.8, which could be due to higher barrier for the PS particles carrying positive surface charges to approach the air–water interface due to image charge effect [41,42,43,44]. It is noteworthy that the PS particles with higher zeta potential (*ca.* +50 mV) can be adsorbed to the air–water interface to stabilize aqueous foams and the PS particles carrying PDEA colloidal stabilizer [21,22,23] with lower zeta potential (*ca.* +30 mV) cannot. There are two possible factors for preventing adsorption of the cationic PS particles carrying PDEA colloidal stabilizer. (1) Higher repulsion between the particles and the air–water interface due to higher image charge effect compared to the amphoteric PS particles synthesized in this study. The PDEA colloidal stabilizer could carry a larger amount of positive charges on the particles. (2) The entropic effect of the PDEA colloidal stabilizer on the PS particle surfaces. The entropies of the PDEA colloidal stabilizer (in other words, the number of possible conformations of the PDEA colloidal stabilizer) decrease if the PS particles carrying PDEA hairs become close to the air–water interface, which is unfavorable from the aspect of the Gibbs free energy. The height of the foam layer gradually decreased until day 16 because of water drainage at pH 2.0 and 4.8. On the other hand, at pH 11.1 the planar aqueous dispersion-air interface appeared just after preparation and the foam layer height was determined to be 0 cm. In the basic condition, the PS particle surfaces are negatively charged and electrostatic repulsion between the air–water interface and the particles avoid the adsorption of the particles to the interface.

The foams consisted of bubbles and their shapes at pH 2.0 and 4.8 were near- and non-spherical, which was revealed by the OM studies, and aspect ratios were in the range between 1.01 and 1.38 (Figure 2b,c). Heywood diameters of the bubbles were estimated to be 219 ± 148 μm and 218 ± 167 μm, respectively. After the application of light pressure on the wet bubbles on the slide glass, the bubbles were ruptured and the inner air came out, which strongly indicates the air bubbles were coated by the PS particles (Figure 2b,c insets).

**Figure 3:**
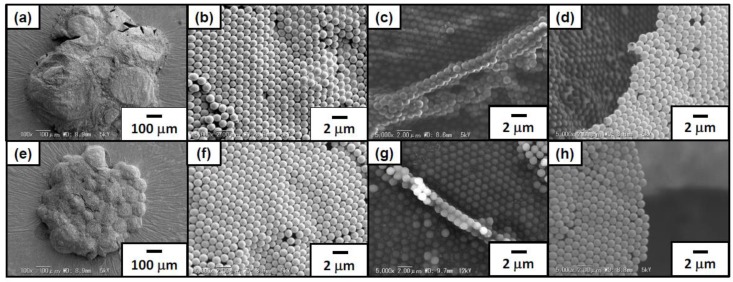
Scanning electron microscope (SEM) images of dried foams stabilized with ACMPA-PS particles prepared at (**a**–**d**) pH 2.0 and (**e**–**h**) pH 4.8. (**b**,**f**) are magnified images of (**a**,**e**). (**c**,**d**,**g**,**h**) are cross-section SEM images of the foams after deliberate rupture using a razor blade.

**Figure 4:**
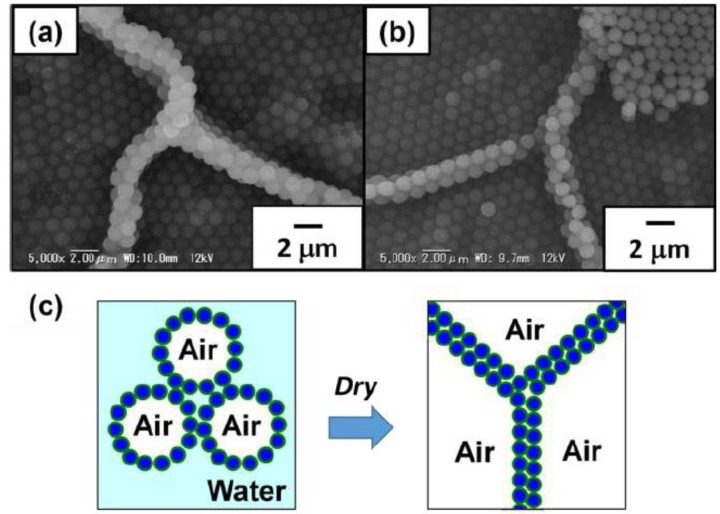
Cross-sectional SEM images of dried foams stabilized with ACMPA-PS particles prepared at (**a**) pH 2.0 and (**b**) pH 4.8 after rupturing using a razor blade. (**c**) Scheme illustrating formation of bilayers of ACMPA-PS particles array via drying.

SEM studies were conducted on the foams prepared from aqueous dispersions with pH 2.0 and 4.8 after removal of free PS particles by washing with deionized water to characterize microstructure of the foams (Figure 3). After drying the continuous water phase overnight at ambient temperature from the aqueous foams, solid foams which kept a three-dimensional structure were obtained (Figure 3a,e). Polydisperse bubbles agglomerated and were deformed due to capillary force without coalescence during/after drying. On the top surface of these dried foams, almost perfect hexagonal-close-packed arrays of the PS particles were observed as reported previously (Figure 3b,f) [11,12]. SEM studies on the dried foam ruptured using a razor blade confirmed the formation of PS particle array bilayers (Figure 3c,g). These particle bilayers should be formed by the contact of bubbles during water drainage from the drying foams, which strongly indicates that the PS particles were adsorbed to the wet bubble surfaces as a monolayer (Figure 4). Based on the mechanism, bilayer junctions were formed from three bubbles. Some PS particle monolayers were also observed, which should be the top layer of the dried bubble, which face to the air phase (Figure 3d,h). Similar results were reported previously [12]. It is noteworthy that flocculated PS particles were hardly observed on the bubble surfaces at pH 4.8, where the PS particles tend to form flocs. There is a possibility that weakly flocculated PS particles might detach from the bubble surfaces due to water flow generated during the evaporation of water and the particles directly adsorbed at the air–water interface could remain on the bubble surfaces.

**Figure 5:**
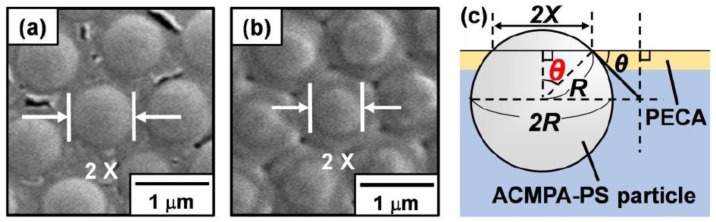
SEM images of ACMPA-PS particles prepared at (**a**) pH 2.0 and (**b**) 4.8 trapped with poly(ethyl 2-cyanoacrylate) (PECA) films recorded from the air-exposed side of the films. (**c**) Determination of the contact angle (through water) of the ACMPA-PS particle at the air–water interface using SEM images.

The position of the PS particles at air-water interface was visualized to estimate the contact angles *θ* (Figure 5) using superglue method [30]. Ethyl 2-cyanoacrylate vapor was introduced to particle monolayer at the air–water interface, which was prepared by autonomous adsorption of the particles via gentle magnetic stirring (Supporting Information). An anionic polymerization at the air–water interface led to the formation of poly(ethyl 2-cyanoacrylate) (PECA) film which trapped the particles at their equilibrium position at the interface. Spherical cap array of the PS particles was observed on the air phase-exposed side of the film and spherical PS particle array was observed on the water-exposed side, which was revealed by SEM studies. The contact angles *θ* of the PS particles measured through the aqueous phase were estimated to be 56° and 41° at pH 2.0 and 4.8, using the average diameters of spherical caps and particles [45]. The *θ* values were estimated to be less than 90°, which indicates that aqueous bubbles were preferably formed rather than liquid marbles [2,3,4,5]. Adsorption energies of the PS particle at the air-water interface (Δ*G*) can be estimated to be 2.88 × 10^6^ and 9.30 × 10^5^ *k*_B_*T* at pH 2.0 and 4.8 using the calculated *θ* values based on Equation (3) [46]:

(3) $$\Delta G=-\gamma_{aw}\pi a^{2}{\left(1-\cos\theta \right)}^{2}$$

where *γ*_aw_ is the surface tension of water, *a* is the particle radius and *k*_B_ is the Boltzmann constant and *T* is temperature.

**Figure 6:**
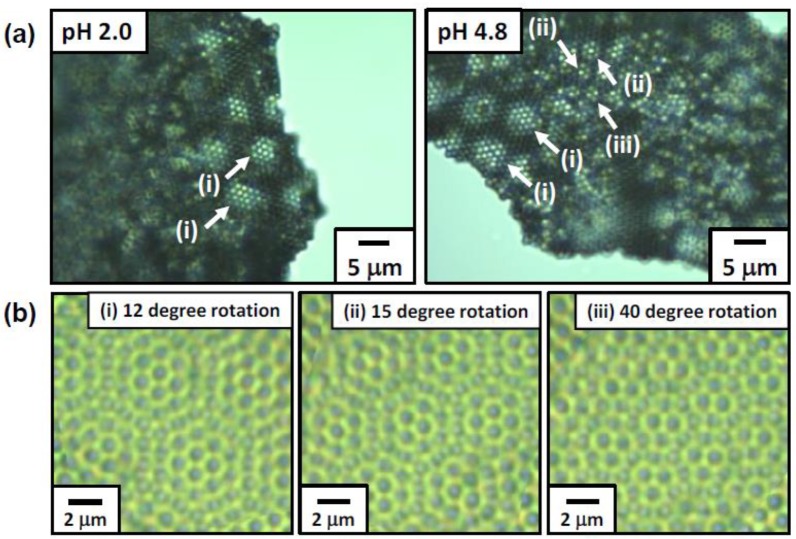
(**a**) Moiré patterns produced by ACMPA-PS particle bilayers as observed by optical microscopy. (**b**) Simulation images obtained by superimposing a semi-transparent (40% transmission) optical micrograph image of a hexagonally ordered particle array onto the identical non-transparent image after rotation of the semi-transparent image through angles of 15, 40 and 50° relative to the non-transparent image.

**Figure 7:**
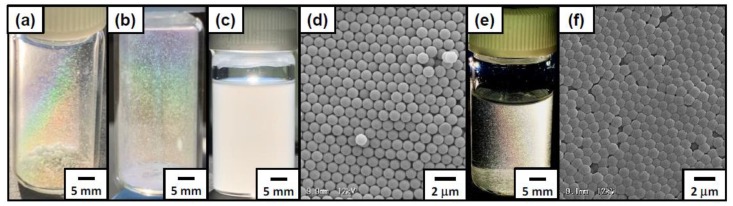
Digital photographs of ACMPA-PS particle-stabilized foam fragments prepared at pH 2.0 viewed under (**a**,**c**,**e**) white light and (**b**) sunlight. The fragments were dispersed in ethanol (**c**) before and (**e**) after annealing with toluene vapor for 15 min. SEM images of foam fragments (**d**) before and (**f**) after the annealing with toluene vapor.

Dried foams were easily broken into small fragments using the razor blade. Due to the PS particle array bilayer, moiré patterns were formed, which could be observed on the crushed dried foams by OM (Figure 6a). The moiré patterns are known to be formed when regular two-dimensional geometric patterns are overlapped with each other [47,48], and here this optical phenomenon occurred by overlapping the two-dimensional highly ordered arrays of hexagonally packed PS particles formed on the bubble surfaces. This optical effect has been observed previously for the particle array bilayer [11,12,49,50,51]. The moiré patterns observed in this study can be simulated by superimposing two identical images of PS particle arrays (the upper image is made semi-transparent) using Microsoft PowerPoint 2013 software. As Figure 6b indicates, the similar moiré patterns with those observed in the OM studies can be produced in an artificial manner by rotating the two images some degrees with respect to each other within the same plane. Furthermore, it is noteworthy that the foam fragments showed an iridescent character under white light and sunlight (Figure 7a,b and Figure S3, Supplementary Materials). Generally, structural color can be observed from colloidal particle arrays consisting of near-monodispersed submicrometer-sized particles [52,53,54,55,56], whose sizes are comparable to the visible light wavelength. Structural colors have been also observed from the micrometer-sized particle arrays [57,58] and the structural color generation mechanism should be the same with that of the foam fragments consisting of micrometer-sized particles, although the clear mechanism is under a veil.

The colloidal array structure of foam fragments could be stabilized via exposure to toluene vapor for 15 min by partially fusing particles with each other (Figure 7d,f). The toluene-treated foam fragments were dispersed in ethanol in their form, resulting in ethanol that was iridescent under strong white light (Figure 7e). On the other hand, the dried foam fragments without toluene treatment were redispersed in ethanol to be a turbid milky-white dispersion (Figure 7c).

## 4. Conclusions

Monodispersed and micrometer-sized amphoteric PS particles carrying amidino and carboxyl groups on their surfaces were successfully synthesized by soap-free emulsion polymerization. It has been demonstrated that the PS particles with cationic and neutral surface charges at pH 2.0 and 4.8 could be adsorbed to the air–water interface to stabilize aqueous foams and those with negative charges at pH 11.1 tend to disperse in aqueous medium resulting in low foamability. The PS particles formed colloidal arrays on the bubble surfaces at pH 2.0 and 4.8, and the dried foams showed moiré patterns due to the formation of particle array bilayers. Moreover, the fragments of dried foams showed an iridescent character under white light. The pH-dependent formation of aqueous foams/bubbles using the polymer particles studied in this study should help in understanding the stability of foams/bubbles utilized in industry including food, textile, petroleum, cosmetic, pharmaceutical and personal care product sections [2,59,60,61,62].

## Supplementary Materials

The following are available online at https://www.mdpi.com/2073-4360/12/3/511/s1: Figure S1: (a) SEM image of ACMPA-PS particles synthesized by soap-free polymerization at pH10. (b) Particle size distribution curve measured by the laser diffraction method., Figure S2: (a) Plots of sediment height (*H*_s_) versus time during the sedimentation of ACMPA-PS particles at pH 2.0, pH 4.8, and 11.1., Figure S3: Digital photographs of fragments of ACMPA-PS particle-stabilized foam prepared at pH 4.8, viewed under (a) white light and (b) sunlight.
