# Supplementary material for: pH-Dependent Foam Formation Using Amphoteric Colloidal Polymer Particles
Source: Polymers (Basel). 2020 Feb 27;12(3):511. doi: 10.3390/polym12030511 (PMC7182924; doi:10.3390/polym12030511)
Supplement: Supplementary file 1 [file polymers-12-00511-s001.pdf]

Supporting Information

pH-dependent foam formation using amphoteric colloidal polymer particles

Sayaka Fukui [1], Tomoyasu Hirai [1,2], Yoshinobu Nakamura [1,2] and Syuji Fujii [1,2,*]

[1] Department of Applied Chemistry, Faculty of Engineering, Osaka Institute of Technology 5-16-1 Omiya, Asahi-ku, Osaka 535-8585, Japan

[2] Nanomaterials Microdevices Research Center, Osaka Institute of Technology 5-16-1 Omiya, Asahi-ku, Osaka 535-8585, Japan

* Correspondence: syuji.fujii@oit.ac.jp; Tel.: +81-(0)6-6954-4274 (Sy.F.)

Zeta potential

Zeta potentials were calculated from the electrophoretic mobility, measured using a Malvern Zetasizer Nano ZS with a MPT-2 Multi-Purpose Titrator. Measurements were conducted as a function of pH with diluted dispersions by gradually adding NaOH, starting from an initial pH of approximately 3. Zeta potentials were averaged over 3 runs at each pH. The variance was typically smaller than 3 mV.

Interfacial particle trapping method

Air-water interfacial particle trapping was conducted following the method established by Vogel et al. (Vogel, N.; Ally, J.; Bley, K.; Kappl, M.; Landfester, K.; Weiss, C. K. Direct visualization of the interfacial position of colloidal particles and their assemblies. *Nanoscale* **2014**, *6*, 6879–6885.). Aqueous dispersion of PS particles (concentration, 5 wt%) placed in a petri dish was magnetically stirred at 350 rpm for 10 min. The pH of the dispersion was tuned to be 2.0 and 4.8 using HCl aqueous solution, respectively. The ethyl 2-cyanoacrylate monomer (0.7 g) was placed in the other petri dish on a hot plate (50 °C). Both petri dishes were placed in a closed container for 30 min. The monomer can evaporate and polymerize at the air–water interface. The anionic polymerization of cyanoacrylate is initiated at the interface upon contact with water, and the polycyanoacrylate is generated. The polymerization reaction of cyanoacrylate is initiated by nucleophiles (*e.g.* water molecules). More monomer is supplied via the gas phase, the polymerization proceeds to eventually cover the air-water interface, embedding particles at the air-water interface in their equilibrium position.

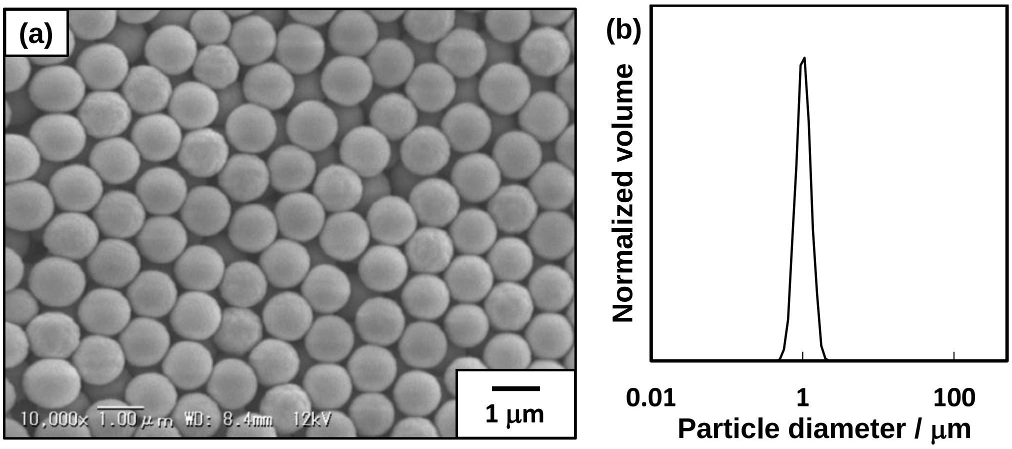

Figure S1 (a) SEM image of ACMPA-PS particles synthesized by soap-free emulsion polymerization at pH10. (b) Particle size distribution curve measured by laser diffraction method.

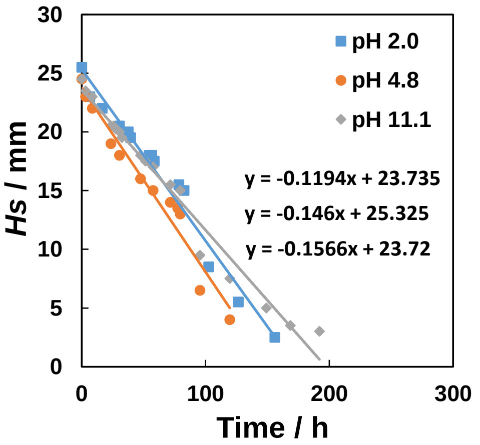

Figure S2 Plots of sediment height (*Hs*) versus time during the sedimentation of ACMPA-PS particles at (■) pH 2.0, (●) pH 4.8, and (♦) 11.1.

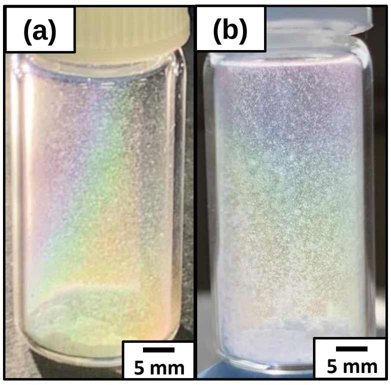

Figure S3 Digital photographs of fragments of ACMPA-PS particle-stabilized foam prepared at pH 4.8, viewed under (a) white light and (b) sunlight.
